# Enhancement of critical heat flux in nucleate boiling of nanofluids: a state-of-art review

**DOI:** 10.1186/1556-276X-6-415

**Published:** 2011-06-09

**Authors:** Hyungdae Kim

**Affiliations:** 1 Department of Nuclear Engineering, Kyung Hee University, Yongin, Gyunggi 446-701, Republic of Korea

## Abstract

Nanofluids (suspensions of nanometer-sized particles in base fluids) have recently been shown to have nucleate boiling critical heat flux (CHF) far superior to that of the pure base fluid. Over the past decade, numerous experimental and analytical studies on the nucleate boiling CHF of nanofluids have been conducted. The purpose of this article is to provide an exhaustive review of these studies. The characteristics of CHF enhancement in nanofluids are systemically presented according to the effects of the primary boiling parameters. Research efforts to identify the effects of nanoparticles underlying irregular enhancement phenomena of CHF in nanofluids are then presented. Also, attempts to explain the physical mechanism based on available CHF theories are described. Finally, future research needs are identified.

## Introduction

Nanofluids are a new class of nanotechnology-based heat-transfer fluids, engineered by dispersing and stably suspending nanoparticles (with dimensions on the order of 1-50 nm) in traditional heat-transfer fluids. The base fluids include water, ethylene, oil, bio-fluids, and polymer solutions. A variety of materials are commonly used as nanoparticles, including chemically stable metals (e.g., copper, gold, silver), metal oxides (e.g., alumina, bismuth oxide, silica, titania, zirconia), several allotropes of carbon (e.g., diamond, single-walled and multi-walled carbon nanotubes, fullerence), and functionalized nanoparticles.

Nanofluids originally attracted great interest because of their abnormally enhanced thermal conductivity [1]. However, recent experiments have revealed additional desirable features for thermal transfer. Key features of nanofluids that have thus far been discovered include anomalously high thermal conductivity at low nanoparticle concentrations [2,3], a nonlinear relationship between thermal conductivity and concentration for nanofluids containing carbon nanotubes [3], strongly temperature-dependent thermal conductivity [4], and a significant increase in nucleate boiling critical heat flux (CHF) at low concentrations [5,6]. State-of-the-art reviews of major advances on the synthesis, characterization, thermal conductivity, and single-phase and two-phase heat transfer applications of nanofluids can be found in [7-17]. However, the available reviews have paid much more attention to thermal properties and single-phase convective heat transfer than to two-phase heat transfer, and even reviews including two-phase heat transfer have only briefly touched upon important new research on the significant increase of CHF in nanofluids.

This paper presents an exhaustive review and analysis of CHF studies of nanofluids over the past decade. The characteristics of CHF enhancement in nanofluids are systemically reviewed according to the effects of boiling parameters. Efforts to reveal the key factors leading to nanofluid CHF enhancement are summarized. Attempts to understand the precise mechanism of the phenomenon on the basis of existing CHF theories are also presented. Finally, future research needs are identified in the concluding remark.

## CHF enhancement in nanofluids

You et al. [5] first demonstrated that when a nanofluid is used instead of pure water as a coolant, CHF can be significantly enhanced. Their test results for pool boiling of alumina-water nanofluid showed that the CHF increased dramatically (approximately 200% increase) at low concentrations (less than 0.01 vol.%) compared with pure water. Significant enhancement of CHF was further confirmed for SiO_2 _particles in water by Vassallo et al. [6]. However, the causes of CHF increases in nanofluids could not be explained using traditional CHF correlations. Since the publication of these pioneering works, extensive experimental studies have been conducted in this area over the past decade. Studies of CHF increase in nanofluids are summarized in Tables 1 and 2 according to pool and flow conditions, respectively.

**Table 1:** Summary of studies on CHF of nanofluids in pool boiling

Reference	Nanofluids	Concentration	Test heater	CHF enhancement
[5]	Al_2_O_3 _in water	0.001-0.025 g/l	Cu plate (10 × 10 mm^2^)	200%, (19.9 kPa)
[6]	SiO_2 _(15, 50, 3,000 nm) in water	0.5 vol.%	NiCr wire (*ϕ *= 1 mm)	60%
[72]	Al_2_O_3 _(38 nm) in water	0.037 g/l	Ti layer on glass	70%
[45]	TiO_2 _(27, 85 nm) in water	0.01-3 vol.%	Cu plate	50%
[22]	Al_2_O_3 _(70-260 nm), ZnO in water; Al_2_O_3 _in ethylene glycol	-	Cu plate	200%
[47]	Al_2_O_3 _(47 nm) in water	0.5-4 vol.%	SS plate (4 × 100 mm^2^)	50%
[73]	Gold (3 nm) in water, 2.3 kPa	-	Cu disk (1 cm^2^)	180%
[32,33]	SiO_2 _(10-20 nm) in ionic solution of water	0.5 vol.%	NiCr wire (*ϕ *= 0.32 mm)	220-320%
[18,53,59,60]	TiO_2 _(23 nm)	10^-5^-10^-1 ^vol.%	NiCr wire (*ϕ *= 0.2 mm)	100%
	Al_2_O_3 _(47 nm) in water		Ti wire (*ϕ *= 0.25 mm)	80%
	SiO_2 _(10 nm)			170%
[46,55]	Al_2_O_3 _(110-210 nm)	10^-3^-10^-1 ^vol.%	SS wire (*ϕ *= 0.381 mm)	50%
	ZrO_2 _(110-250 nm) in water			75%
	SiO_2 _(20-40 nm)			80%
[20]	CuO (30 nm) in water	0.1-2.0 wt.%	Cu plate (40 × 40 mm^2^); with grooves	50%, (100 kPa)
				140%, (31.2 kPa)
				220% (7.4 kPa)
[57]	Al_2_O_3 _(45 nm) in water and ethanol	0.001-10 g/l	Glass, Au, and Cu surfaces	40%
[21]	CuO (59 nm) and SiO_2 _(35 nm) in water and alcohol (C_2_H_4_OH) with SDBS surfactant	0.2-2 wt.%	Cu disk (*ϕ *= 20 mm)	30%
[19]	Al_2_O_3 _(22.6, 46 nm) in water	0.0006-0.01 g/l	NiCr wire (*ϕ *= 0.64 mm)	50%
	BiO_2 _(38 nm)			33%
[23]	Al_2_O_3 _(<25 nm) in water	10^-4^-10^-1 ^g/l	Cu disk (*ϕ *= 10 and 15 mm)	70%
	Ag (3, 10, 80, 150, 250 nm)			35%
[35]	Single-walled CNT in water with hydrochloric acid	2 wt.%	NiCr wire (*ϕ *= 0.32 mm)	300%
[74]	Multi-walled CNT in water with PVP polymer	10^-4^-10^-2^, 0.05 vol.%	Cu plate (9.5 × 9.5 mm^2^) Ti wire (*ϕ *= 0.25 mm)	200% (19.9 kPa) 140% (19.9 kPa)
[36]	Cu (10-20 nm) in water	0.25, 0.5, 1.0 wt.%	Plate (30 × 30 mm^2^)	
	w/ SDS surfactant			50%
	w/o SDS surfactant			-30%
[69]	TiO_2 _(45 nm) and Al_2_O_3 _(47 nm) in water	0.01 vol.%	Cu and Ni disks (*ϕ *= 20 mm)	40%
[28,75,76]	Al_2_O_3 _(139 nm), CuO (143 nm), Diamond (86 nm) in water	0.001-1 g/l	Cu plate (10 × 10 mm^2^)	80%
[27]	CNT in water with nitric acid for pH 6.5;	0.5-4 wt.%	Cu plate (40 × 40 mm^2^)	60% (100 kPa)
				140% (31.2 kPa)
				200% (7.4 kPa)
[63]	Graphene in water	0.001 vol.%	NiCr wire	84%
	Graphene-oxide in water			179%
	Al_2_O_3 _in water			152%

**Table 2:** Summary of studies on CHF of nanofluids in flow boiling

Reference	Nanofluids	Concentration	Test conditions	CHF enhancement
[38,77,78]	Al_2_O_3 _(40-50 nm) in water	10^-3^-10^-1 ^vol.%	SS316 tube (5.45 and 8.7 mm I.D.)	53%
	ZrO_2 _(50-90 nm)		1,000-2,500 kg/m^2^s	53%
	Diamond (4 nm)		Inlet subcooling: <20 K	38%
[39]	Al_2_O_3 _(50 nm) in water	10^-3^-0.5 vo.l%	SS316 tube (11 mm I.D.)	70%
			100-300 kg/m^2^s	
			Inlet subcooling: 25 and 50 K	
[40,41]	Al_2_O_3 _(47 nm) in water	0.01 vol.%	Rectangular channel (10 × 5 mm^2^)	40%
			1-4 m/s	
			Inlet subcooling: 0 K (saturated)	
			Single side heating: Cu disk (*ϕ *= 10 mm)	
[42]	Al_2_O_3 _(25 nm) in water	10^-3^-10^-1 ^vol.%	SS tube (*ϕ *= 510 μm)	50%
			600-1,650 kg/m^2^s	
			Inlet temperature: 30-404C	

In this section, characteristics of CHF enhancement in nanofluids that have been identified from an exhaustive review of published studies over the past decade will be summarized in terms of the effects of primary parameters as follows:

1. nanoparticle concentration,

2. nanoparticle material and size,

3. heater size,

4. system pressure,

5. existence of additives, and

6. flow conditions.

### Influence of nanoparticle concentration

**Figure 1:**
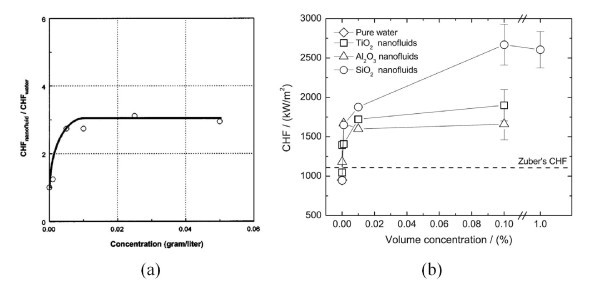
**Effect of nanoparticle concentration on CHF enhancement in nanofluids**. (**a**) Al_2_O_3_-water nanofluid on flat Cu plate with 10 × 10 mm^2 ^area [5]; (**b**) various nanofluids on NiCr wire with 0.2-mm diameter [18].

CHF enhancement in nanofluids is strongly dependent on nanoparticle concentration. Figure 1 shows the experimental results of You et al. [5] and Kim et al. [18] for the CHF of nanofluids in pool boiling, which was investigated by varying the nanoparticle concentration over a wide range from 10^-5 ^to 10^-1 ^vol.%. Increasing the nanoparticle concentration increased the CHF continuously up to a certain concentration, and thereafter, the CHF remained more or less constant at the maximum enhancement value. This nanoparticle concentration vs. enhancement trend was further confirmed by the experimental studies of Golubovic et al. [19] and Liu et al. [20,21], although their quantitative values differed because of discrepancies in experimental parameters, such as the shape of the heater and the nanoparticle material. Thus, it is reasonable to examine the effects of various boiling parameters in terms of the maximum CHF value.

### Influence of nanoparticle material and size

**Figure 2:**
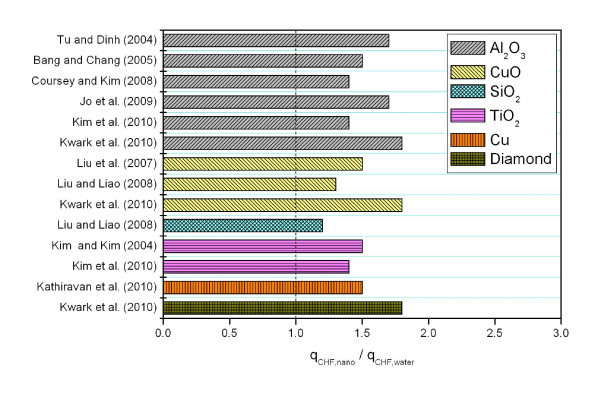
**The CHF increase in nanofluids with different nanoparticles on flat plates**.

Material and size are important properties influencing the characteristics of nanoparticles. The choice of nanoparticles to be suspended in a base fluid is expected to have an essential influence on the maximum possible increase in CHF. Figure 2 shows the increase in CHF for different nanofluids from selected studies in Table 1, all having water without additive as the base fluid, and all tested with flat-plate heaters. Even for the same nanoparticle material, considerable data scatter was observed, presumably due to variations in the dispersion status of the particles and the geometry of the heaters used in the tests.

**Figure 3:**
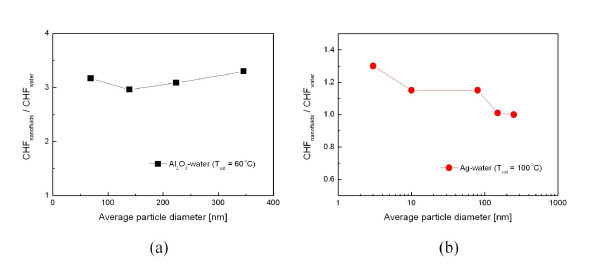
**Effect of nanoparticle size on CHF enhancement in nanofluids**. (**a**) [22]; (**b**) [23].

Moreno et al. [22] examined the size dependence of alumina-water nanofluid CHF using gravimetrically separated nanofluids with average particle diameters of 69, 139, 224, and 346 nm. They found that the magnitude of CHF enhancement was nearly identical for each nanofluid sample under saturated pool-boiling conditions at a concentration of 0.025 g/l (see Figure 3). Recently, Jo et al. [23] investigated the size effect using silver nanoparticles with mean particle diameter ranging from 3 to 250 nm. In contrast to Moreno et al. [22]'s results, the greatest increase (approximately 31%) in CHF occurred for the nanofluid with 3-nm particles, and the enhancement decreased with increasing particle size. In summary, it is not possible to draw any conclusions on the effects of nanoparticle material and size from an analysis of the existing data. More systematic studies must be carried out to clarify the effects of nanoparticle material and size on CHF enhancement in nanofluids.

### Influence of heater geometry

**Figure 4:**
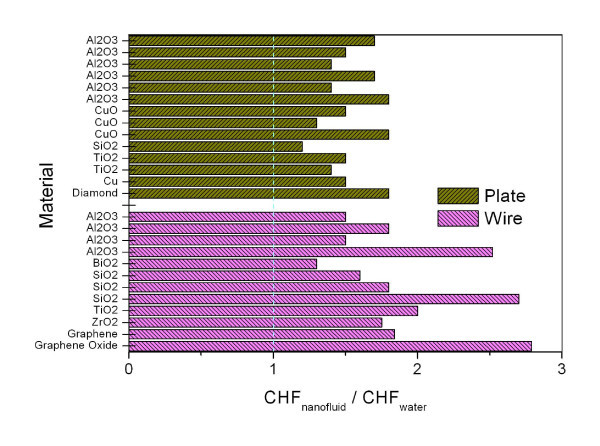
**Experimental results of measured CHF values for both flat plates and thin wires**. All are under atmospheric condition and with no additive.

Nucleate boiling experiments for studying the CHF of nanofluids are normally conducted with thin wires or flat plates. Many previous studies used thin wires as a boiling surface to confirm an intriguing feature of nanofluids during nucleate boiling: significant CHF increase compared with a reference value for pure water. Thin wires were used to simplify the measurement of average heat flux and surface temperature and the post-inspection of the heater surface. However, the measured CHF values might be different from those obtained with the flat plates used in general applications. Figure 4 summarizes the experimental results for both flat plates and thin wires, all under atmospheric conditions and with no additive. A comparison of the CHF values for the two different heater geometries reveals that CHF enhancement is greater with thin wires (50 to approximately 200%) than with flat plates (30 to approximately 80%). This difference in the measured CHF values is due to the different CHF mechanisms with thin wires and large flat plates. Nucleate boiling with flat plates proceeds to film boiling via the hydrodynamic CHF mechanism, whereas CHF with thin wires is caused by the local dryout mechanism governed by boiling incipience phenomena, provided that hydrodynamic instabilities are absent [24].

**Figure 5:**
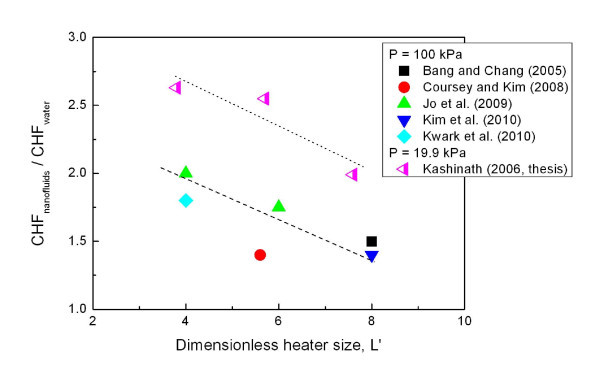
**Relation between characteristic size of flat-plate heater and maximum CHF enhancement in Al_2_O_3_-water nanofluids**.

From the point of view of understanding the general characteristics of CHF enhancement in nanofluids, the experimental results obtained with flat plates are more reliable than those obtained with thin wires. Thus, to infer the general effect of heater size from previous studies, the maximum CHF enhancements of alumina-water nanofluids on flat-plate heaters exclusively are plotted against the dimensionless heater size *L*',(1)

where *L, ρ, σ*, and *g *are the characteristic heater size, fluid density, surface tension, and gravitational acceleration, respectively. The resulting plot is given in Figure 5. It is shown that expansion of the heating area in the range of L' from 4 to 8 diminishes the CHF enhancement of nanofluids. Even though all the data are obtained on the flat plate, the values of *L*' are still in the range where CHF of pure fluid is strongly dependent upon the size of heating surfaces [25]. Hamamura and Kato [26] explained that an inflow of liquid from the surrounding, instead of the top, increases CHF on a finite flat-plate-type heater and this effect is stronger on a smaller heater. In this range of *L*', the impact of nanofluids on CHF is likely dependent upon different flow characteristics around the heating surfaces. Experiments are needed to confirm this so that the CHF enhancement of nanofluids in many high-flux systems with different characteristic dimensions could be predicted accurately.

### Influence of pressure

Pressure affects nucleate boiling heat transfer and CHF by influencing physical properties such as the vapor density, latent heat of vaporization, and surface tension of the working fluids. Liu et al. [20,27] investigated the effect of system pressure on the CHF enhancement of nanofluids, including those with alumina nanoparticles and carbon nanotubes. They found that CHF enhancement in nanofluids is a strong function of system pressure and the enhancement effect is more significant at lower pressures. This discovery is consistent with the system pressure vs. CHF trend of the experimental results obtained by You and his coworkers [5,22,28,29] with an identical heater geometry and experimental setup.

**Figure 6:**
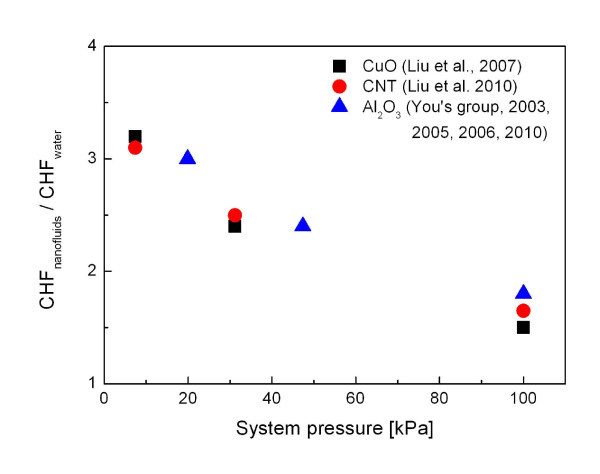
**Effect of pressure on the maximum CHF enhancement in nanofluids**. The used heater geometries are 40 × 40 mm^2 ^[20,27] and 10 × 10 mm [5,22,28,29].

Figure 6 shows the pressure dependency of CHF enhancement in nanofluids. It is of interest that the CHF enhancement apparently decreases with increasing the pressure. This pressure effect cannot be simply explained by traditional boiling CHF theory, but however, some insight can be given based on a comparison of behaviors of dry patches, whose irreversible growth can cause CHF [26,30], under different pressure conditions. Van Ouwerkerk [31] found that when the CHF is approached, the mechanism of formation of dry areas is different for atmosphere and low-pressure conditions: the large dry patch is created by coalescence of small vapor bubbles that forms at atmospheric pressure but underneath are individual bubbles growing to immense size at low pressure. This different mechanism of formation of dry patches under atmospheric and low-pressure conditions suggests that the pressure in nanofluid boiling can have strong impact on the CHF enhancement. In addition, if the use of nanofluids alters local properties of individual bubbles growing on the heating surface, such as wetting ability, its impact on the CHF value can be more significant in low-pressure condition where a dry patch underneath a single bubble plays a key role in triggering CHF.

### Influence of additive

Ionic additives and surfactants can significantly distort the nucleate boiling heat transfer and CHF phenomena in nanofluids by influencing the stability of the particles and their mutual interactions near the heated surface. Kumar and his coworkers [32-35] primarily investigated the effects of ionic additives. Their experimental results demonstrated that when the surface tension of a nanofluid is carefully controlled with ionic additives such as HCl and NaOH, its performance can be further intensified, resulting in a CHF nearly three or four times higher than that of pure water. On the other hand, Kathiravan et al. [36] conducted pool-boiling CHF experiments on Cu-water nanofluids with and without sodium lauryl sulfate (SDS) anodic surfactant. Although the nanofluid without surfactant exhibited CHF increases of up to 50% (which is consistent with the results of previous studies), the CHF of the nanofluid with surfactant was severely diminished, presumably due to the reduction in surface tension. In conclusion, previous studies reveal that the effect of additives such as ionic additives and polymer surfactants on the CHF performance of nanofluids can be strong, but our current understanding of the effect is very limited. Additional research will be required to understand the role of additives in the nucleate boiling heat transfer and CHF of nanofluids.

### Influence of flow condition

Although most CHF experiments with nanofluids have been carried out under pool-boiling conditions, there have been a very limited number of CHF studies in forced convection condition. A group at MIT (USA) reported for the first time that nanofluids can significantly enhance the CHF under subcooled flow boiling conditions [37,38]. They conducted subcooled flow boiling experiments in a stainless steel tube with an internal diameter of 8.7 mm at a pressure of 0.1 MPa for three different mass fluxes (1,500, 2,000, and 2,500 kg/m^2 ^s). The maximum CHF enhancements were 53%, 53%, and 38% for nanofluids with alumina, zinc oxide, and diamond, respectively, all obtained at the highest mass flux. Kim et al. [39] performed similar flow boiling CHF experiments in a stainless steel tube with an internal diameter of 10.98 mm at relatively low mass fluxes ranging from 100 to 300 kg/m^2 ^s and inlet subcooling temperatures of 25°C and 50°C. The results for alumina nanofluids confirmed a significant flow boiling CHF enhancement of up to about 70% under all experimental conditions.

Later, a group at POSTECH (South Korea) investigated the flow boiling CHF of nanofluids under saturated conditions [40,41]. To visualize liquid-vapor two-phase structures in nanofluid flow boiling, they used a rectangular channel made of transparent strengthened acryl with a cross-sectional area of 10 × 5 mm (width × height). The working fluid was heated only on a short-heated surface (a disk with a diameter of 10 mm) placed at the bottom of the flow channel, and a maximum CHF enhancement of 40% was achieved. It was reported using the visualization results that the existence of nanoparticle deposition alters the wetted fraction of the heating surface by cooling liquid under forced convection, delaying the occurrence of the CHF.

Recently, some research tried to assess feasibility of the use of nanofluids for small-sized cooling systems utilizing flow boiling heat transfer. Vafaei and Wen [42] investigated subcooled flow boiling of alumina-water nanofluids in small single circular microchannels with a diameter of 510 μm and reported an increase of approximately 51% in the CHF at 0.1 vol.%. On the other hand, in similar experiments conducted by Lee and Mudawa [43] with alumina-water nanofluids at 1.0 vol.%, the CHF point could not be reached due to severe clogging of the circular flow channel (500 μm diameter). Obviously, good stability of nanoparticles in nanofluids is a critical requirement for application to cooling systems with small flow channels.

## Investigations to find key factors of CHF enhancement in nanofluids

All the experimental studies listed in Tables 1 and 2 have produced some enhancement in CHF under both pool and flow boiling conditions. To account for the observed phenomena, all probable factors associated with nanoparticles have been thoroughly examined, focusing on the physical properties of nanofluids and nanoparticle-surface interactions. In this section, these investigations and the resulting advances are reviewed to understand the key factors responsible for the increased CHF of nanofluids.

### Physical properties of nanofluids

The application of nanofluids to boiling heat transfer was first motivated by their abnormally enhanced thermal conductivity at nanoparticle concentrations on the order of a few percent by volume [44]. However, You et al., in their pioneering research [5] on CHF enhancement in nanofluids, reported that continued increases in CHF were not observed at concentrations higher than approximately 0.01 vol.%, which is significantly lower than the usual concentration of nanoparticles used for the enhancement of thermal conductivity in nanofluids. Thus, the observed CHF increases could not be explained in terms of the effect of nanoparticles on thermal conductivity enhancement. In addition to thermal conductivity, it was revealed that all other physical properties of dilute nanofluids, including surface tension, vapor and liquid density, viscosity, heat of vaporization, and boiling point temperature, are almost the same as the corresponding properties of pure water [28,45,46]. In summary, the transport and thermodynamic properties of nanofluids at low concentration (<0.01 vol.%) are very similar to those of pure water. It can be concluded that changes in the properties of nanofluids do not account for the enhancing effect of nanoparticles on liquid-to-vapor phase-change heat transfer.

### The two underlying roles of nanoparticles during boiling

To interpret the mechanism of CHF enhancement in nanofluids, two kinds of hypotheses on the roles of nanoparticle during nanofluid boiling were suggested in the early stage of research.

**Figure 7:**
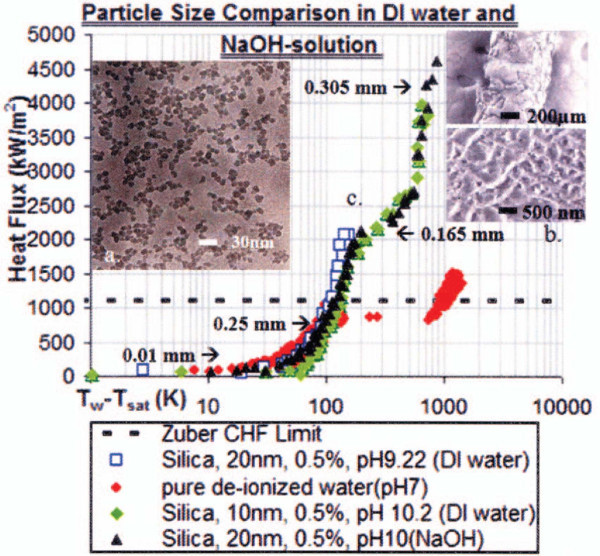
**SEM picture of NiCr wire after deposition of silica nanoparticles during nanofluid boiling **[32].

Vassallo et al. [6] (one of the initial studies in which significant CHF enhancement in nanofluids was observed) reported that a major deposition of nanoparticles (about 0.15-0.2 mm thick) occurs on the heater surface during nanofluid boiling, suggesting some possible interactions between the nanoparticles and the heated surface at high heat fluxes. Soon afterward, Milanova and Kumar [32] and Bang and Chang [47] confirmed that nanoparticles precipitate on the surface during nucleate boiling, forming a layer whose morphology depends on the nanoparticle material, and suggesting some surface effects on CHF phenomena such as the trapping of liquid near the heater surface due to porous structures and the breakup of voids near the surface. Figure 7 shows a SEM picture of NiCr wire after deposition of silica nanoparticles during nanofluid boiling.

**Figure 8:**
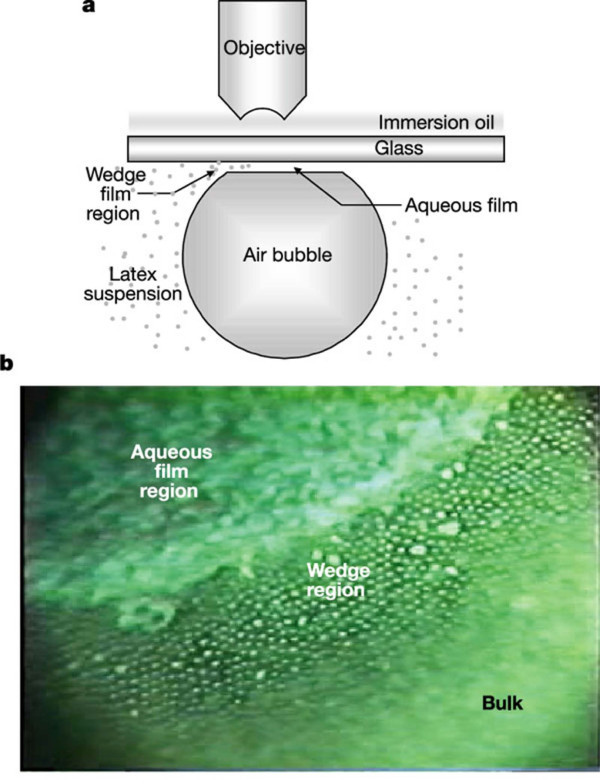
**Ordered layering of nanoparticles in the confined wedge of the evaporating meniscus**. (**a**) Diagram of experimental setup. (**b**) Particle structuring in a wedge film [49].

Sefiane [48] suggested an alternative approach to clarify the mechanism by which the presence of nanoparticles affects heat transfer and CHF during boiling. He demonstrated experimentally that the nanoparticles in the liquid promote the pinning of the contact-angle line of the evaporating meniscus and sessile drops. He explained that the observed results were due to the structural disjoining pressure stemming from the ordered layering of nanoparticles in the confined wedge of the evaporating meniscus [49] (Figure 8) and suggested that an analysis of the boiling heat transfer of nanofluids could account for the strong effect of nanoparticles on the contact-line region via the structural disjoining pressure. Wen [50,51] subsequently carried out further investigations of the influence of nanoparticles on the structural disjoining pressure. He calculated the equilibrium meniscus shape in the presence of nanoparticles and found that the vapor-liquid-solid line could be significantly displaced toward the vapor phase by the presence of nanoparticles in the liquid. He therefore concluded that the structural disjoining pressure caused by nanoparticles can significantly increases the wettability of the fluids and inhibits the development of dry patches, triggering CHF.

**Figure 9:**
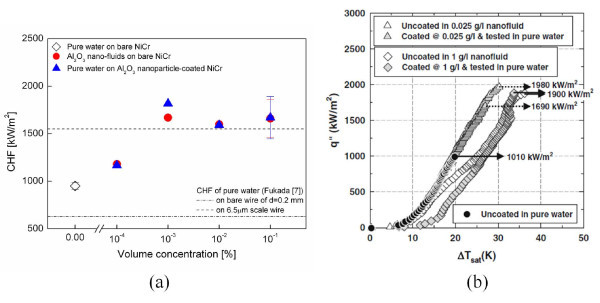
**Effect of nanoparticle layer in alumina-water nanofluids**. (**a**) [53]; (**b**) [28].

The above-described two effects of nanoparticles (i.e., modification of the heater surface and structural disjoining pressure) both seem to be plausible hypotheses for CHF enhancement in nanofluids. However, to understand the principle mechanism of the phenomena, it is necessary to examine the single contribution of each factor to the enhanced CHF performance of nanofluids. Kim et al. [52,53] carried out an insightful experiment to separate the single effect of the nanoparticle deposition layer on the CHF of nanofluids. First, they conducted a pool-boiling test of a nanofluid using a fresh heater wire. A subsequent surface inspection confirmed the presence of a nanoparticle deposition layer on the heater wire. They then performed an additional CHF test on the nanoparticle-deposited wire submerged in pure water, which resulted in a CHF enhancement of the same magnitude as that of the nanofluids. The experimental results clearly demonstrated that the enhancement of CHF in nanofluids is due to the modification of surface topology associated with nanoparticle deposition on the heater surface during nanofluid boiling. Moreover, Golubovic et al. [19] and Kwark et al. [28] recently conducted the same experiments using both thin wire and flat-plate heaters and obtained experimental results consistent with those of Kim et al. [52,53]. Figure 9 shows the experimental results obtained by Kim et al. [53] and Kwark et al. [28].

The preceding conclusion on the role of nanoparticle deposition is compatible with the recent work of Kim et al. [54], who studied pool boiling heat transfer during the quenching of a hot sphere in a nanofluid. They reported that the CHF remained unchanged when a clean sphere was cooled in the nanofluid, and it was only enhanced during the cooling of a sphere with a nanoparticle layer. This result, therefore, confirmed that the deposition layer of the nanoparticles plays a critical role in effectively enhancing the CHF by modifying the heater surface. In conclusion, an understanding of the underlying mechanism should be sought to study the influence of the nanoparticle-deposited surface on CHF.

### The nanoparticle layer on the surface

**Figure 10:**
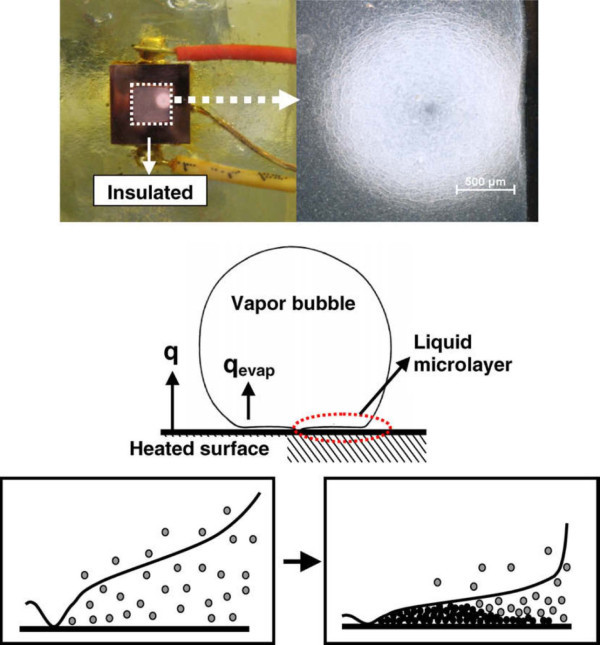
**Images of nanparticle coating generated, on the heater surface **[28].

Before proceeding to the assessment of surface effects on CHF enhancement in nanofluids, a prior question arises: why are nanoparticles deposited on the heater surface during nucleate boiling of nanofluids? Kim et al. [52] reported that the nanoparticle layer developed only during nucleate boiling in nanofluids, but was not caused by gravitational sedimentation or single-phase natural convection. Kim et al. [46] suggested the hypothesis that the evaporation of microlayers initially containing nanoparticles could be the reason for the formation of the porous layer. As vapor bubbles grow, the evaporating liquid leaves behind nanoparticles, which then concentrate at the base of the bubbles, forming the microlayer. As the microlayer evaporates, nanoparticles are again left behind, and they then bond to the hot heater surface. Kwark et al. [28] recently confirmed this theory by optically observing a single circular nanoparticle coating formed on a boiling surface, where a single-active bubble nucleation site was allowed to undergo several boiling cycles, as shown in Figure 10. Accordingly, nanofluid boiling itself, and specifically microlayer evaporation, is responsible for producing the nanoparticle layer on the surface.

**Figure 11:**
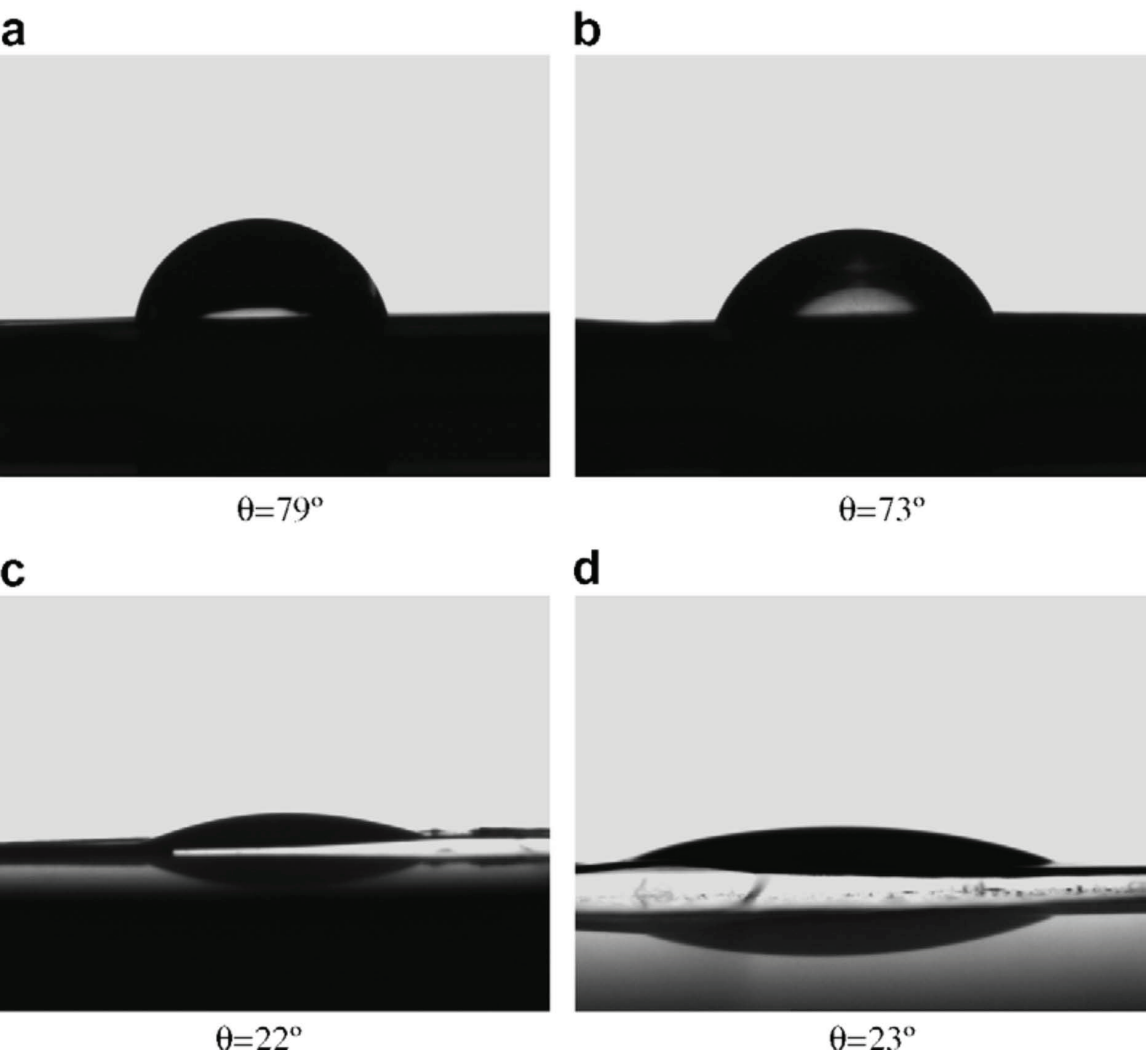
**Static contact angles of 5-μL sessile droplets on stainless steel surfaces**. (**a**) Pure water droplet on surface boiled in pure water, (**b**) alumina nanofluid droplet on surface boiled in pure water, (**c**) pure water droplet on surface boiled in alumina nanofluid, (**d**) alumina nanofluid droplet on surface boiled in alumina nanofluid [46].

Kim et al. [46,55] investigated the surface effect on CHF enhancement of water-based nanofluids containing alumina, zirconia, and silica nanoparticles. In their research, the deposition of nanoparticles on the heater surface significantly improved the wettability, as measured by the reduction of the static contact angle (see Figure 11). Note that no appreciable differences were found between pure water and nanofluids. They inferred that the buildup of a porous layer with oxide nanoparticles increases the adhesion tension *γ*_SV _- *γ*_SL _and the roughness factor *r *(the ratio of the effective contact area to the smooth contact area), and both effects lead to a pronounced reduction of the contact angle in accordance the modified Young-Laplace equation [56],(2)

where *θ *and *σ *are the contact angle and the surface tension, respectively. A systematic review of the prevalent CHF theories then demonstrates that higher wettability can produce a CHF enhancement consistent in magnitude with the experimental observations of numerous researchers. Subsequently, a number of studies focusing on the role of a nanoparticle layer, including Coursey and Kim [57], Liu and Liao [21], Golubovic et al. [19], and Jeong et al. [58], produced the same conclusions as Kim et al. [46] in regard to the significant improvement of surface wettability and its role in the CHF enhancement of nanofluids.

**Figure 12:**
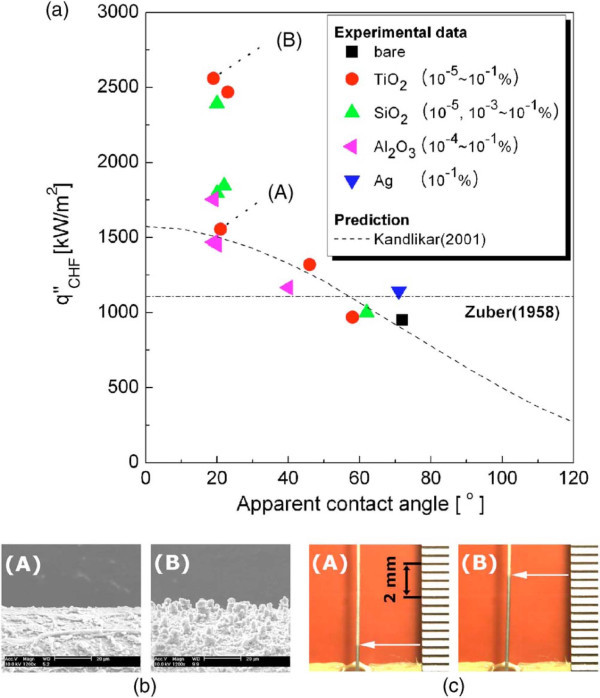
**Relation between CHF and surface characteristics**. (**a**) CHF of pure water vs. contact angle of a water droplet on nanoparticle-deposited surfaces. SEM pictures (**b**) and maximum capillary wicking height of pure water (**c**) for surfaces boiled in 10^-3^% (A) and 10^-1^% (B) water-TiO_2 _nanofluids, with the same contact angles of ~20° [60].

Kim et al. [59] also found that a porous layer of nanoparticles significantly improved the surface wettability. On the other hand, they were the first to show that the effect of wettability alone cannot explain additional increases beyond the attainable CHF value when the contact angle approaches zero. By focusing on the role of capillarity in the CHF behavior of nanofluids, they showed that capillarity causes the liquid to rise on the nanoparticle-deposited surfaces in accordance with(3)

where *R*_c _and cos*θ *represent the microscopic structures and surface wettability of the nanoparticle layers, respectively. Capillary flow during boiling supplies fresh liquid to the dry region beneath the vapor bubbles, delaying the irreversible growth of hot spots and CHF. Kim and Kim [60] used capillarity to characterize a completely wetted nanoparticle-coated surface. They showed that the estimated heat-flux gain due to capillary liquid supply along the porous layer was of the same order of magnitude as that due to wettability enhancement (Figure 12). They concluded that the significant CHF enhancement of nanofluids during pool boiling is a consequence not only of increased surface wettability, but also of improved capillarity resulting from the surface deposition of nanoparticles.

A consensus explanation of the cause of CHF enhancement in nanofluids seems to be obtainable via an intense study focused on the effect of the nanoparticle layer. In other words, the CHF of a nanofluid is enhanced by its improved ability to actively wet the heater surface, thanks to the porous structure of the thin nanoparticle sorption layers.

## Exploration of the mechanism of CHF enhancement in nanofluids

Several recent studies have tried to link existing CHF theories and experimental results in nanofluids, for example, Kim et al. [46] and Golubovic et al. [19], to incorporate the improved surface properties caused by nanoparticle deposition. However, owing to the complexity of the phenomenon of CHF and to numerous factors affecting it, a sufficiently definite theory by which the nanoparticle layer results in such a high CHF enhancement has not yet emerged. According to the extensive reviews by Kim et al. [46] and Golubovic et al. [19], most hypotheses fall into one of the following four categories:

1. hydrodynamic instability model,

2. macrolayer dryout model,

3. bubble interaction model,

4. hot/dry spot model.

In this section, the previous studies aiming to understand the physical mechanism of CHF enhancement in nanofluids in terms of the above predominant CHF theories is reviewed.

### Hydrodynamic instability model

The hydrodynamic instability model of Zuber [61] is the most widely used correlation to predict pool boiling CHF,(4)

where *h*_fg _is latent heat of evaporation. However, the initial research of the nanofluid CHF (for example, You et al. [5] and Kim et al. [46]) discarded hydrodynamic instability theory as an interpretative tool for CHF in nanofluids because of its inability to account for surface effects. In fact, the CHF correlation of Eq. 4 does not depend on the fluid properties at all, whereas the primary reason for increased CHF in nanofluids is the change in surface characteristics associated with the deposition of nanoparticles during nanofluid boiling. You et al. [5] concluded using their nanofluid CHF studies that some important unknown factors, potentially missing from Zuber's theory, might be responsible for the increased CHF in nanofluids.

On the other hand, Golubovic et al. [19] suggested a possible approach to interpreting the CHF mechanism in nanofluids by modifying the hydrodynamic instability models of Lienhard and Dhir [25] and Ramilison et al. [62]. They hypothesized that a change in the surface contact angle alters the size and spacing of the vapor jets above the heater surface, so that the surface effect on the nanoparticle deposition can be incorporated into the hydrodynamic CHF model proposed by Lienhard and Dhir [25].

Recently, Park et al. [63] reported that the CHF of water-based nanofluids containing graphene/graphene-oxide nanoparticles was as high as that of alumina nanofluid, even though neither the wettability nor the capillarity of the surface was improved on the nanoparticle layers. Alternatively, they measured the dewetting wavelengths of water on heater wires and reported that the wavelength change corresponds to the CHF enhancement tendency for all tested nanofluids. Although a direct correlation between the critical instability wavelength obtained from Zuber's theory and the dewetting wavelength of the liquid is questionable, they concluded that the wavelength modulation most adequately supports the CHF enhancement of nanofluids.

### Macrolayer dryout model

Haramura and Katto [26] proposed the macrolayer dryout model. In this model, CHF occurs due to macrolayer dryout if the heat flux is sufficient to evaporate the macrolayer before the departure of the mushroom bubble. Kim et al. [46] examined the impact of the improved wettability of a nanoparticle layer (or reduction of the contact angle) on the equivalent thickness of the macrolayer. When the macrolayer thickness was calculated using the model of Sadasivan et al. [64], they found that a contact angle reduction due to nanoparticle deposition could produce an increase in the thickness of the liquid layer enough to result in a significant increase in the CHF.

### Bubble crowding model

Bubble crowding at a heated surface was proposed by Rosenhow and Griffith [65]. In this model, close packing of bubbles on the heater surface is responsible for the cessation of the liquid flow toward the surface, leading to CHF. More sophisticated theories include the effect of the shear force generated by the mutual interaction of growing and departing bubbles, for example, Kolev [66],(5)

where Δ*τ_W _*and Δ*τ_d _*are the bubble wait time and departure time at the heated surface, respectively. In Eq. 5, the bubble departure time is a strong function of the nucleation site density (*n*"). According to the Wang and Dhir [67] correlation, the site density decreases with the contact angle. Thus, the intensity of the shear stress generated by the mutual interactions of bubbles grows slowly on a heated surface with a low contact angle compared with a surface with a large contact angle. Kim et al. [46] found that according to the Kolev [66] model, a change in the contact angle *θ *can have a major impact on the CHF. Therefore, it could be concluded that the bubble-interaction theory supports the notion of surface wettability improvement as a plausible cause of CHF enhancement in nanofluids.

### Hot/dry spot model

Hot spot model was first proposed by Unal et al. [68]. This model suggests that the temperature at the center of a dry patch on the heater surface is an important parameter that can trigger CHF. Ability of cooling liquid to rewet the heated dry area should make the strong impact on CHF. In accordance with this idea, Theofanous and Dinh [30] proposed the modified hot spot model with focus on the micro-hydrodynamics of the solid-liquid-vapor line at the boundary of a hot/dry spot. In their model, CHF occurs when the evaporation recoil force (which drives the liquid meniscus to recede) becomes larger than the surface tension force (which drives the meniscus to advance and rewet the hot/dry spot).

Kim et al. [46] semi-quantitatively showed that the improved wettability on the nanoparticle-fouled surface significantly increases the surface tension force to rewet the hot/dry spot, suggesting higher CHF. In this regard, they concluded that the hot/dry spot model incorporating the micro-hydrodynamics of a liquid meniscus corroborates the link between increased wettability and CHF enhancement in nanofluids. In addition, Kim et al. [69] conducted sessile-drop wetting experiments focused on the effect of a nanoparticle layer on the stability of an evaporating meniscus. They found that an individual liquid meniscus is more stable on an alumina nanoparticle layer and hence can sustain the evaporation recoil force at a higher heat flux. The evaporative heat-flux gain attainable on the nanoparticle layer was of the same order of magnitude as the CHF increases in nanofluids. Thus, these experimental results also supported the hot/dry spot theory based on the micro-hydrodynamics of a liquid meniscus.

Several recent studies have demonstrated that the CHF model proposed by Kandlikar and Steinke [70] is reasonably well correlated with measured CHF data in nanofluids as a function of contact angle (see, for example, [40,71]). This model utilizes the evaporation momentum force and receding contact angle *β *as parameters. Fundamentally, this model also focuses on the micro-hydrodynamics of the vapor-liquid interface of a single bubble at the heater surface based on the force balance at the solid-vapor-liquid triple-contact line. The accuracy of this model in predicting the CHF of nanofluids supports the argument that the hot/dry spot model incorporating the micro-hydrodynamics of an evaporating meniscus is a plausible mechanism.

## Concluding remarks

Over the past decade, a considerable amount of research has been carried out in the area of nucleate boiling critical heat flux (CHF) in nanofluids. It is now known that in both pool and flow boiling, the CHF capability of conventional heat transfer fluids (such as water or alcohol) is significantly improved by suspending nanoparticles in the base liquids even at small particle concentrations (less than 0.1 vol.%).

The present review of available studies indicated that there is a general consensus in the key cause of CHF enhancement in nanofluid boiling: the thin nanoparticle layer formed on the heater surface, during nucleate boiling of nanofluids, increases the CHF via their improved ability to wet the heater surface. Although appropriate modifications of all the traditional CHF theories succeed in demonstrating approaches to and possibilities for incorporating the impact of microscale deposition of nanoparticles with nanoscale pores, a sufficiently definite theory to link the improved wettability and the increase of CHF on the nanoparticle layer has not yet emerged owing to the complexity of the phenomenon of CHF and to the lack of information about microscale two-phase flow underneath bubbles. It is very difficult to figure out the underlying mechanism leading to CHF from on relatively large-scale conventional nucleate boiling experiments, which only yield time- and space-averaged information of the complex phenomenon of CHF. In this regard, to understand the fundamental mechanism of CHF enhancement in nanofluids, the efforts by researchers have to focus on obtaining the full details of two-phase heat transfer near the heater surface (for example, direct measurement of the time-dependent temperature and liquid-vapor phase distributions on the heater surface in high heat-flux nucleate boiling).

Another area that merits further study is the effect of pressure and heater geometry. A systematic review of available data in literature revealed that the magnitude of the CHF enhancement in nanofluids is very strongly dependent on system pressure and heater geometry. These parametric effects must be carefully considered when assessing the potential of nanofluids for various industrial applications. For example, it is doubtable if nanofluids significantly enhance CHF even in the high-pressure condition, such as nucleate reactor core. Experiments are needed to extend the nanofluids' usability to many high-flux systems with a wide diversity of heater geometry and pressure conditions.

From a practical point of view, considering application of nanofluids to actual thermal-flow systems, good stability of nanoparticles is one of the critical necessary conditions, as indicated in the review of the microchannel flow boiling applications. Adding ionic additive to control electrostatic condition of solution is one of the simplest options to improve dispersion stability of nanoparticles in nanofluids, but it can severely alter characteristic structures of nanoparticle deposition on a heater surface, resulting in the distorted nucleate boiling CHF performance. There is no systematic study available in literature that describes the effects of additives on nucleate boiling CHF in nanofluids.

## Competing interests

The author declares that they have no competing interests.

## Authors' contributions

HK conducted the extensive literature review and drafted the manuscript. The author read and approved the final manuscript.
